# Platelet is the early predictor of bronchopulmonary dysplasia in very premature infants: an observational cohort study

**DOI:** 10.1186/s12890-022-01895-2

**Published:** 2022-03-27

**Authors:** Xiaoling Wang, Yan Ma, Shenghui Wang, Wenbin Dong, Xiaoping Lei

**Affiliations:** 1 Division of Neonatology, Department of Pediatrics, The Affiliated Hospital of Southwest Medical University, No. 8, Section 2, Kangcheng Road, Luzhou, 646000 China; 2 Sichuan Clinical Research Center for Birth Defects, No. 8, Section 2, Kangcheng Road, Luzhou, 646000 China

**Keywords:** Bronchopulmonary dysplasia, Platelet parameters, Premature infant, Thrombocytopenia

## Abstract

**Background:**

A previous study showed that the lungs are involved in the biogenesis of platelets (PLTs). Thus, the present study aimed to investigate the association between bronchopulmonary dysplasia (BPD), a chronic lung disease, and PLT parameters in very premature infants.

**Methods:**

The study subjects were premature infants with a gestational age of ≤ 30 weeks and birth weight of ≤ 1500 g in a preterm birth cohort study recruited between January 1, 2015, and August 31, 2019. BPD was defined as the need for oxygen supplementation more than 28 days after birth. The PLT count, mean platelet volume (MPV), platelet distribution width (PDW), and plateletcrit (PCT) level were compared between BPD and non-BPD infants. A generalized estimating equation model was used to adjust for confounding factors. A forward stepwise logistic regression model was used to calculate the adjusted odds ratio (OR) for thrombocytopenia in the BPD group. Receiver operating characteristic curve analysis was performed to assess the predictive value of PLT count combined with gestational age (GA) and birth weight (BW) for BPD.

**Results:**

The final study subjects were 134 very premature infants, namely, 64 infants with BPD and 70 infants without BPD. The BPD infants had lower PLT counts (*F* = 4.44, *P* = 0.03) and PCT levels (*F* = 12.54, *P* = 0.00) than the non-BPD infants. However, the MPV (*F* = 14.25, *P* = 0.00) and PDW (*F* = 15.04, *P* = 0.00) were higher in the BPD group. After adjusting for potential confounding factors, the BPD infants had a higher risk of thrombocytopenia than the non-BPD infants (adjusted aOR 2.88, 95% CI 1.01–8.15), and the risk of BPD was increased in very premature infants with a PLT count ≤ 177*10^9^/L (OR 4.74, 95% CI 1.93–11.62) at the end of the second week. In the multivariate predictive model, it was showed that the AUC area (0.85), sensitivity (0.88), specificity (0.70) and Youden index (0.58) are improved using PLT counts ≤ 177*10^9^/L combined with GA and BW.

**Conclusions:**

Abnormal PLT parameters were observed in BPD infants, and a PLT count ≤ 177*10^9^/L was a potential risk factor for the development of BPD in very premature infants.

**Supplementary Information:**

The online version contains supplementary material available at 10.1186/s12890-022-01895-2.

## Background

Bronchopulmonary dysplasia (BPD) is the most common respiratory complication of very-low-birth-weight (VLBW) and very premature infants [1]. Over the last few decades, advancements in respiratory support strategies and the use of pulmonary surfactants have significantly increased the survival rate of VLBW and very premature infants. However, an increasing number of survivors suffer from BPD [2] and BPD-related mortality and morbidity, including a predisposition to chronic respiratory and cardiovascular impairment, growth failure, and neurodevelopmental delays [3, 4]. The main pathological characteristic of BPD is the arrest of lung development, which manifests as a decrease in the alveolar number, an increase in alveolar volume, simplification of the alveolar structure, and abnormal pulmonary microvascular morphology [5].

Neonatal thrombocytopenia is a common hematological abnormality in newborn infants, particularly in premature infants [6]. Generally, the mechanism of thrombocytopenia in premature infants involves multiple factors, including prematurity, infections (sepsis, necrotizing enterocolitis [NEC]), and asphyxia [7]. In a previous study [8], Lefrançais E et al. showed that the lungs were involved in the biogenesis of platelets (PLTs) and could produce approximately 50% of total PLTs or 10 million PLTs per hour. Based on these findings, the authors proposed that the lung was a primary site of terminal PLT production and a potentially important hematopoietic organ. Therefore, the present study hypothesized that the abnormalities of pulmonary lesions and microvascular morphology in developing and developed BPD may affect the production and release of PLTs in the lung. Therefore, PLT parameters in the peripheral blood may be changed before and/or after BPD diagnosis and may be used to predict the risk of BPD in very premature infants.

## Methods

A nested case–control study was conducted in very premature infants recruited in a preterm birth cohort from January 1, 2015, to August 31, 2019. Written informed consent was accepted from the guardian while the infants were enrolled in the cohort. The present study is the secondary analysis of the cohort study and the protocol was reviewed and approved by the institutional research ethics committee of the Affiliated Hospital of Southwest Medical University.

### Inclusion criteria

(1) Gestational age (GA) at birth ≤ 30 weeks, (2) birth weight ≤ 1500 g, (3) admission to the hospital within 24 h after birth, and (4) hospital stay more than 28 days.

### Exclusion criteria

(1) Severe congenital constructional and/or chromosome malformations, such as congenital heart or lung disease, central nervous system malformation, diaphragmatic hernia, congenital deformity of the head, face or thorax, genetic metabolic disease, or chromosomal disease; (2) hematological disease or pulmonary disease; or (3) death in the first 7 days of life.

**Fig. 1:**
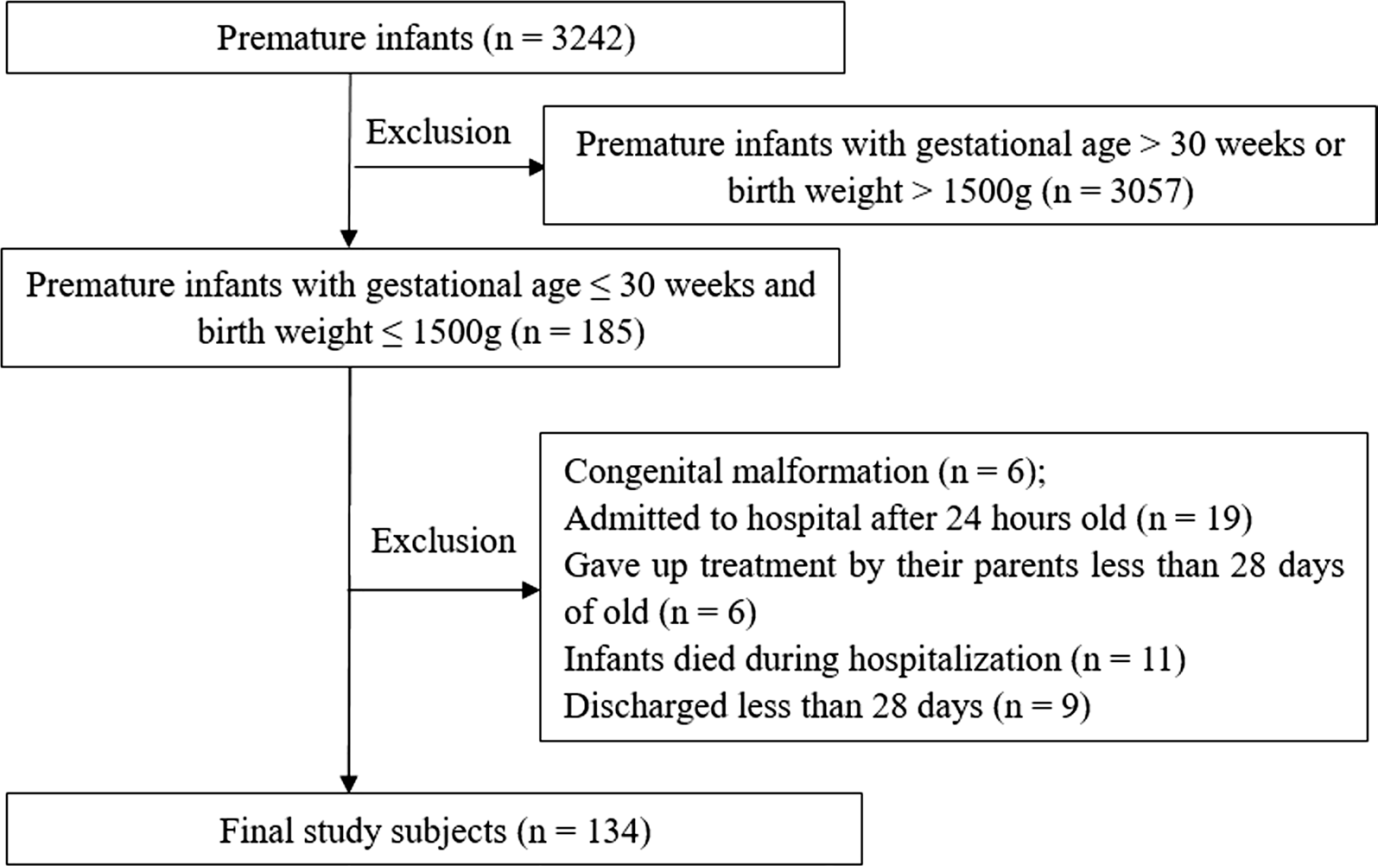
Flow chart of patient screening

A total of 3,242 preterm infants were recruited in the preterm birth cohort, 185 of whom had a GA ≤ 30 weeks and a birth weight ≤ 1500 g, and all were admitted to the neonatal intensive care unit. Infants with congenital malformations (n = 6) or admission exceeding 24 h after birth (n = 19) were excluded. Infants with a hospital stay less than 28 days, including 11 infants who died and 15 infants discharged after recovery, were also excluded. The study subsequently enrolled 134 infants, including 64 infants in the BPD group and 70 infants in the non-BPD group (Fig. 1).

### Diagnostic criteria

According to the diagnostic criteria of the National Institute of Child Health and Human Development (NICHD), BPD was defined as a need for supplemental oxygen for 28 days and was categorized at 36 weeks postmenstrual age (PMA) or 56 days of age for infants > 32 weeks GA as “mild, moderate, or severe” based on oxygen use and/or respiratory support [9]. The patients were subsequently divided into the BPD group and the non-BPD group.

Thrombocytopenia was defined as a PLT count < 150 × 10^9^/L confirmed by peripheral blood smears [10]. Severe thrombocytopenia was defined as < 50 × 10^9^/L [11]. As thrombocytopenia in the first several days is more related to early onset infection, thrombocytopenia after the first week of life was used as an outcome in the present analysis.

### Data collection

A uniform questionnaire was used to extract the medical data of the eligible premature infants from the hospital information system. Epidata was used to record data by two researchers independently to ensure the accuracy of the data. Inconsistent data were assessed by a third researcher.

The potential risk factors and confounders were as follows.

1. Maternal factors: premature rupture of membranes > 18 h, gestational diabetes mellitus, gestational hypertension, antenatal use of glucocorticoids and magnesium sulfate, chorioamnionitis, systemic antibiotics 48 h before delivery, infection during pregnancy, and thrombocytopenia during pregnancy.
2. Infantile factors: GA, sex, birth weight, Apgar score at one minute and five minutes after birth, mode of delivery, presence of infection at admission, presence of intrauterine growth restriction, pulmonary surfactant replacement therapy, mechanical ventilation support and lasting time, nasal continuous positive airway pressure (nCPAP), noninvasive ventilator support and lasting time, reuse of noninvasive ventilator or invasive ventilator, duration of oxygen supplementation, and maximum oxygen fraction use.
3. Blood cell counts: Based on the clinical guidelines of our department, the first blood cell counts were measured at patient admission. During hospitalization, blood cell counts were performed weekly. Tests for infection were performed immediately if infection was suspected. The tests were performed with an XS-800i automatic blood cell analyzer. The PLT count, mean platelet volume (MPV), plateletcrit (PCT) level, and platelet distribution width (PDW) data were extracted and used as outcomes in the analysis.

### Statistical analysis

Statistical analysis was performed with SPSS 21.0 software. The normality of the continuous data was tested. Normally distributed variables are presented as the mean ± standard deviation (*Mean* ± *SD*), and abnormally distributed data are presented as the median and quartile range. Comparisons of the continuous data were made using an independent sample *t*-test or rank sum test. Categorical data are presented as the frequency and percentage and were compared by means of a chi-square test. Multivariate forward stepwise logistic regression was used to calculate the odds ratio (OR) of thrombocytopenia in the BPD group and adjust for potential confounders (*P* < 0.15 for inclusion, *P* > 0.1 for exclusion). The PLT parameters were subsequently compared between the BPD and non-BPD groups at each time point (two-way ANOVA). A generalized estimating equation (GEE) model for repeated measurement data was established to examine the effects of the interaction between BPD and age on the PLT parameters. The GEE model was also used to adjust for infection status or other potential confounding factors. Finally, receiver operating characteristic (ROC) curve analysis was performed to assess the predictive value of PLT count at each time point for BPD. After getting the cut-off value of PLT count, a multivariate ROC curve was used to test whether combining PLT count can improve the predictive value of GA and BW for BPD.

## Results

As shown in Table 1, the infants in the BPD group had smaller GAs, lower birth weights, and lower one-minute and five-minute Apgar scores than the infants in the non-BPD group (*P* < 0.05). The incidence of infection during pregnancy, duration of nCPAP use, reuse of invasive and noninvasive ventilation, duration of invasive ventilation, duration of oxygen supplementation, highest fractional inspired oxygen, and intrauterine growth restriction were higher in the BPD group (*P* < 0.05).

**Table 1:** Comparison of baseline characteristics between the two groups

	BPD group (n = 64)	Non-BPD group (n = 70)	*x*^2^/t/z	*P*
Sex (male/female)	34/30	51/19	5.61	0.02
Gestational age (week)^a^	28 (25, 30)	29 (28, 30)	− 4.28	0.00
Birth weight (g)	1136 ± 194	1311 ± 165	− 5.65	0.00
Maternal infections (yes/no)	23/41	18/52	5.16	0.02
Pregnancy-induced hypertension (yes/no)	3/61	1/69	0.36	0.60
Gestational diabetes mellitus (yes/no)	5/59	9/61	0.91	0.34
Premature rupture of membranes (yes/no)	30/34	30/40	0.22	0.64
Chorioamnionitis (yes/no)	4/60	3/67	0.02	0.90
Antibiotics 48 h before delivery (yes/no)	33/31	37/33	0.02	0.88
Antenatal corticosteroid use (yes/no)	46/18	53/17	4.95	0.18
5-Minute Apgar score^a^	7 (6, 8)	9 (7, 9)	− 3.31	0.00
Mode of delivery (cesarean section/vaginal delivery)	10/54	14/56	0.44	0.51
PS treatment (yes/no)	62/2	68/2	0.00	1.00
CPAP (yes/no)^b^	64/0	69/1		1.00
CPAP duration (days)^a^	13 (6, 26)	6 (3, 9)	− 5.60	0.00
Invasive ventilation (yes/no)	11/53	4/66	4.43	0.04
Duration of invasive ventilation (h)^a^	136 (50, 190)	122 (50, 198)	− 0.13	0.90
Reuse of ventilator (yes/no)^c^	28/36	16/54	4.91	0.03
Duration of oxygen supplementation (days)^a^	35 (27, 54)	17 (7, 27)	− 7.31	0.00
Maximum oxygen concentration (duration > 2 h, %)^a^	28 (25, 35)	25 (23, 30)	− 3.91	0.00
Intrauterine growth restriction (yes/no)	13/51	5/65	4.99	0.03
Maternal thrombocytopenia (yes/no)	1/63	2/68	0.00	1.00

*BPD* bronchopulmonary dysplasia, *CPAP* continuous positive airway pressure

^a^Presented as the median and interquartile range (IQR)

^b^The expected value of two cells was less than 5, and Fisher's exact test was used.

^c^Includes invasive and noninvasive ventilation

There were 45 cases with mild BPD, 17 cases with moderate BPD, and 2 cases with severe BPD. Of the 134 preterm infants, 48 (35.8%) met the definition of thrombocytopenia at least once during hospitalization, and 46 (34.3%) were diagnosed after the first week of life. From the 7th to 28th day of life, the incidence of thrombocytopenia (51.56% vs. 21.43%, *P* < 0.05) and severe thrombocytopenia (12.50% vs. 0%, *P* < 0.05) in the BPD group was higher than that in the non-BPD group. BPD was associated with an increased risk of thrombocytopenia using multiple forward stepwise Logistic regression to adjust for confounders (aOR2.88, 95% CI 1.01–8.15) (Table 2).

**Table 2:** The risks of thrombocytopenia in BPD infants relative to non-BPD infants

Group	Thrombocytopenia (n, %)	OR	95% CI	aOR	95% CI
BPD vs non-BPD	33 (51.56) vs 15 (21.43)	4.42	(2.08, 9.40)	2.88	(1.01, 8.15)
IUGR vs non-IUGR	13 (20.30) vs 5 (7.14)	3.35	(1.20, 9.35)	2.57	(0.88, 7.53)

*BPD* bronchopulmonary dysplasia, *IUGR* intrauterine growth restriction, *aOR* adjusted odds ratio, *CI* confidence interval

BPD and IUGR are included in the final model

As shown in Table 3, in the non-BPD group, PLT counts gradually increased after birth (*F* = 2.53, *P* < 0.05), but there were no significant variations in the BPD group. With the exception of the first day, the PLT counts in the BPD group were lower than those in the non-BPD group (*P* < 0.05 for all)*.* Using the GEE model to adjust for confounding factors, the PLT counts in the BPD group were lower than those in the non-BPD group (*F* = 4.44, *P* = 0.03). The differences in PCT between the two groups were similar to the differences in PLT counts.

**Table 3:** Comparison of the platelet counts and plateletcrit levels of the two groups

Parameters	Age	BPD infants (n = 64)	Non-BPD infants (n = 70)	Each time point (*t*, *P*)	Model (*F*, *P*)	
					ANOVA	GEE^a^
Platelet count	Day 1	247 ± 80	239 ± 76	0.55, 0.58	2.53, 0.04	4.44, 0.03
	Day 7	239 ± 99	276 ± 109	− 2.02, 0.04		
	Day 14	233 ± 116	287 ± 101	− 2.84, 0.00		
	Day 21	220 ± 105	274 ± 133	− 2.56, 0.01		
	Day 28	226 ± 123	274 ± 118	− 2.27, 0.02		
Plateletcrit	Day 1	0.27 ± 0.08	0.29 ± 0.07	− 1.26, 0.21	2.74, 0.03	12.54, 0.00
	Day 7	0.27 ± 0.10	0.32 ± 0.11	− 2.42, 0.02		
	Day 14	0.28 ± 0.11	0.36 ± 0.12	− 4.23, 0.00		
	Day 21	0.27 ± 0.12	0.33 ± 0.13	− 2.89, 0.01		
	Day 28	0.26 ± 0.09	0.33 ± 0.11	− 3.97, 0.00		

*BPD* bronchopulmonary dysplasia, *GEE* generalized estimating equation, *ANOVA* two-way repeated measures analysis of variance

^a^The gestational age, birthweight and infection status were included in the GEE model

The MPV and PDW in the BPD group were higher than those in the non-BPD group at each time point except on the first day (*P* < 0.05 for all)*.* Using the GEE model to adjust for confounding factors, the MPV (*F* = 14.25, *P* = 0.00) and PDW (*F* = 15.04, *P* = 0.00) in the BPD group were higher than those in the non-BPD group (Table 4).

**Table 4:** Comparison of the mean platelet volume and platelet distribution width between the two groups

Parameters	Age	BPD group (n = 64)	Non-BPD group (n = 70)	Each time point (*t*, *P*)	Model (*F*, *P*)	
					ANOVA	GEE^a^
Mean platelet volume	Day 1	10.67 ± 0.92	10.55 ± 0.904	0.75, 0.46	6.93, 0.00	14.25, 0.00
	Day 7	10.80 ± 0.87	10.49 ± 0.82	2.12, 0.04		
	Day 14	11.07 ± 1.13	10.11 ± 0.74	5.89, 0.00		
	Day 21	11.14 ± 0.94	10.77 ± 0.93	2.23, 0.03		
	Day 28	11.17 ± 0.89	10.74 ± 1.02	2.52, 0.01		
Platelet distribution width	Day 1	15.19 ± 3.00	15.13 ± 2.66	0.13, 0.89	2.64, 0.04	15.04, 0.00
	Day 7	14.90 ± 2.61	13.99 ± 2.52	2.03, 0.04		
	Day 14	15.09 ± 2.52	13.53 ± 2.67	3.47, 0.00		
	Day 21	15.31 ± 2.69	14.15 ± 2.80	2.44, 0.02		
	Day 28	15.65 ± 2.05	14.06 ± 2.73	*3.79, 0.00*		

*BPD* bronchopulmonary dysplasia, *GEE* generalized estimating equation, *ANOVA* two-way repeated measures analysis of variance

^a^The gestational age, birthweight and infection status were included in the GEE model

**Fig. 2:**
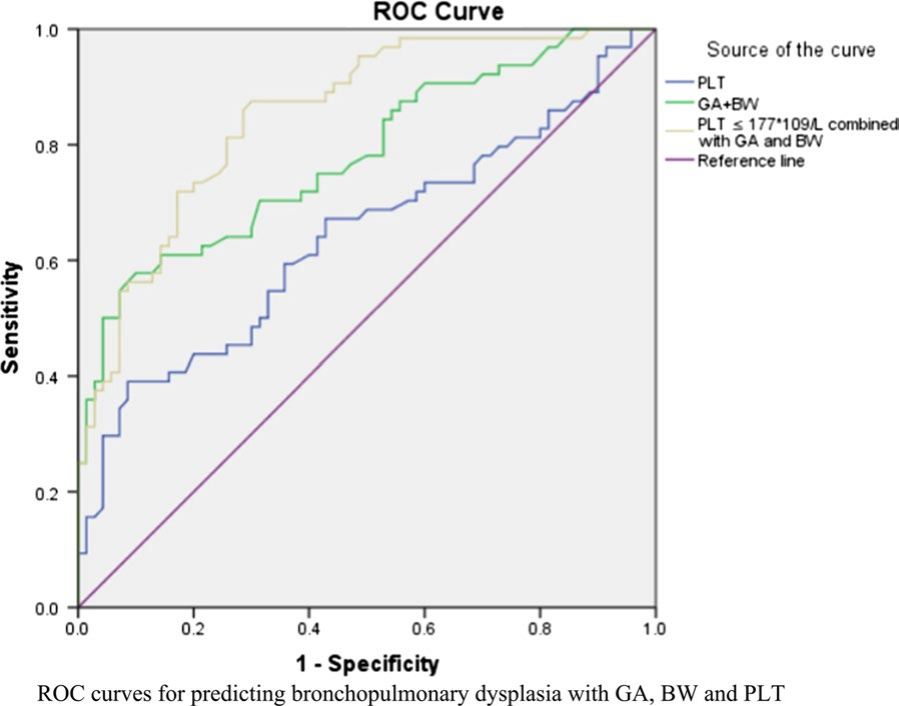
ROC curve for predicting bronchopulmonary dysplasia

The area under the curve (AUC) and its corresponding PLT count at each time point are shown (Additional file 1: Table S1). The results showed that in the second week, a PLT count of ≤ 177*10^9^/L had the best predictive value (AUC 0.63, sensitivity 0.40, specificity 0.88). Compared to those with a PLT count > 177*10^9^/L, infants with a PLT count ≤ 177*10^9^/L in the second week had a significantly higher risk of BPD (OR 4.74, 95% CI 1.93–11.62). Subsequently, in the multivariate predictive model, it was showed that the predictive value for BPD was improved using PLT counts ≤ 177*10^9^/L combined with gestational age and birth weight (Fig. 2 and Table 5).

**Table5:** The predictive value of platelet and its associated factors for BPD

	AUC	Sensitivity	Specificity	Youden Index
PLT	0.63	0.40	0.88	0.29
GA + BW	0.78	0.58	0.90	0.48
PLT ≤ 177*10^9^/L combined with GA and BW	0.85	0.88	0.70	0.58

The PLT, GA and BW are used as continues variable in the bivariate model, and the data-driven cut off value is 177*10^9^/L

## Discussion

In this nested case–control study, BPD was found to be associated with a higher risk of thrombocytopenia and abnormal PLT parameters. The abnormal development of the lungs seems to influence the biogenesis of PLTs, and a PLT count ≤ 177*10^9^/L in the second week of life is a potential risk factor for the development of BPD in very premature infants.

Thrombocytopenia is a common complication in NICUs, with 18% to 35% of patients developing this problem before hospital discharge. Neonatal thrombocytopenia is defined as a PLT count < 150 × 10^9^/L in newborn infants [10–12], and it is more common in extremely low birth weight (< 1000 g) newborns [13]. The prevalence of thrombocytopenia in NICUs is inversely associated with birth weight and GA [14]. The frequencies of thrombocytopenia in preterm neonates with birth weights < 1000 g and < 750 g are reported to be 75% and 90%, respectively [15]. In the present study, the incidence of thrombocytopenia was 35.1%, which is lower than that reported by Del Vecchio A [15]. This difference could be interpreted by the larger gestational weeks and higher birth weight of the premature infants in the present study.

PLTs are usually considered to be small pieces of cytoplasm that disintegrate from the cytoplasm of mature megakaryocytes in bone marrow and are involved in a variety of physiological activities. Megakaryocytes, the parent cells that spawn PLTs, are found in the mammalian lung and in the pulmonary vascular beds. The PLT counts in postpulmonary vessels are significantly higher than those in pulmonary arteries [16]. This indicates that the lung is a site of thrombopoiesis, which refers to the intricate process of PLT production, and that the lungs of humans and other mammals act as reservoirs of PLTs, releasing them in response to certain stimuli [17]. Using state-of-the-art techniques, Lefrançais et al. revealed the lung to be a primary site for PLT biogenesis. Furthermore, the authors quantified the lungs’ contribution to PLT production and found that the contribution was approximately half the total PLT [8]. At the same time, an increasing number of studies on adults have shown that lung injury and lung diseases, such as pulmonary cystic fibrosis, asthma, tuberculosis, and pulmonary hypertension, are associated with a reduction in circulating blood PLTs [18].

BPD is a common chronic inflammatory lung disease of VLBW infants and very preterm infants and is associated with arrested lung development and microvascular dysplasia [5]. Reducing or morphologically altering the pulmonary capillary bed disrupts the pulmonary megakaryocyte fragmentation distribution or number of fragmentation steps. In the present study, in the non-BPD group, the PLT count and PCT increased gradually with increasing age, peaking in the second and third weeks after birth, before decreasing slightly in the following fourth week. However, in the BPD group, the PLT count and PCT did not increase with increasing age and remained at a low level. Furthermore, they were lower than those in the non-BPD group after the first day of life. The PCT level was affected by the PLT number and volume and was positively associated with the PLT count. Our findings showed that the PLT count and the PCT level of the BPD infants were significantly lower than those of the non-BPD group. A previous study reported that PLT parameters on the first day of life were not associated with BPD in preterm infants [19]. Actually, it was consistent with our findings that no differences in PLT parameters were observed on the first day of life between the two groups, and differences existed from the 7th day of life. These findings may further indicate that PLT biogenesis is influenced by the development of BPD. Chronic inflammation in the development of BPD could increase the consumption and destruction of megakaryocytes. At the same time, arrested lung development and microvascular dysplasia of BPD can also influence the biogenesis and release of PLTs, resulting in a reduction in the number of circulating PLTs [20].

MPV, PDW, and PCT level are three common parameters related to PLT function. MPV is a measurement of the average PLT size, and PDW is an indicator of the variation in PLT size. Normally, MPV and PDW are inversely associated with the PLT count [21]. In some adverse conditions, PLTs are destroyed in the peripheral circulation. To compensate for this destruction, the production function of PLTs is stimulated, and many immature PLTs are released into the peripheral circulation. Immature/early mature PLTs contain more protein, enzymes, and granules and have a vigorous metabolism, with higher volumes that show more heterogeneity. Dani C et al. reported a significant difference in the MPV between BPD and non-BPD infants and speculated that BPD was associated with a higher MPV [22]. Cekmez F et al. also found that premature infants with BPD, NEC, and IVH had higher MPV levels in early postnatal life than control infants and showed that this phenomenon was associated with inflammatory and oxidative processes [23]. In the present study, BPD was also found to be associated with elevated MPV and PDW in VLBW and very premature infants. One possible explanation for this is the inflammatory reaction and oxidative stress injury that occurs in the lungs of BPD patients. As BPD develops, certain adverse factors, such as an increased need for mechanical ventilation and oxygen, promote the recruitment of inflammatory cells in pulmonary tissue, and the subsequent enhanced inflammatory reaction may induce a higher MPV and PDW [23].

There are several limitations to the present study. First, although the GEE model was used to adjust for potential confounders, the findings in the present analysis could not totally avoid bias from the differences in GA and birth weight between the two groups. However, in the same conditions, there were no differences in the baseline platelet parameters between the two groups in the first day of life. Thus, we have some confidence that the changes of the platelet parameters in the BPD group after the first week were partly associated with the lung impairment. Second, as an observational study, a causal relationship between BPD and changes in PLT parameters was not confirmed. Third, due to the relatively small size of the study population, it was not possible to perform stratification analysis according to the severity of BPD.

## Conclusion

Abnormal PLT parameters were observed in infants with developing and developed BPD, and a reduce PLT count ≤ 177*10^9^/L as a potential risk factor for the development of BPD.

## Supplementary Information

**Additional file 1.** Table S1 Calculation of cut-off discriminating bronchopulmonary dysplasia status.

## Data Availability

We declare that the materials described in the manuscript, including all relevant raw data, will be freely available from the corresponding author by email to any scientist wishing to use them for noncommercial purposes without breaching participant confidentiality.

## Abbreviations

PLTs: Platelets
PLT: Platelet
BPD: Bronchopulmonary dysplasia
MPV: Mean platelet volume
PDW: Platelet distribution width
PCT: Plateletcrit
OR: Odds ratio
VLBW: Very-low-birth-weight
NEC: Necrotizing enterocolitis
GA: Gestational age
NICHD: The National Institute of Child Health and Human Development
PMA: Postmenstrual age
nCPAP: Nasal continuous positive airway pressure
GEE: Generalized estimating equation
ROC: Receiver operating characteristic
AUC: Area under the curve
