# Supplementary material for: Platelet is the early predictor of bronchopulmonary dysplasia in very premature infants: an observational cohort study
Source: BMC Pulm Med. 2022 Mar 27;22:109. doi: 10.1186/s12890-022-01895-2 (PMC8962022; doi:10.1186/s12890-022-01895-2)
Supplement: Supplementary file 1 — Additional file 1. Table S1 Calculation of cut-off discriminating bronchopulmonary dysplasia status. [file 12890_2022_1895_MOESM1_ESM.docx]

**Table S1:** Calculation of cut-off discriminating bronchopulmonary dysplasia status

|  | AUC | Sensitivity | Specificity | Youden Index | Cut-off value  Platelet count,10^9^/L |
| --- | --- | --- | --- | --- | --- |
| Day 1 | 0.52 | 0.39 | 0.70 | 0.09 | 260 |
| Day 7 | 0.59 | 0.50 | 0.68 | 0.18 | 242 |
| Day 14 | 0.63 | 0.40 | 0.88 | 0.28 | 177 |
| Day 21 | 0.60 | 0.73 | 0.43 | 0.17 | 281 |
| Day 28 | 0.60 | 0.29 | 0.95 | 0.25 | 145 |
